# Estimation of individual beneficial and adverse effects of intensive glucose control for patients with type 2 diabetes

**DOI:** 10.1007/s00125-016-4082-5

**Published:** 2016-09-01

**Authors:** Joep van der Leeuw, Frank L. J. Visseren, Mark Woodward, Yolanda van der Graaf, Diederick E. Grobbee, Stephen Harrap, Simon Heller, Giuseppe Mancia, Michel Marre, Neil Poulter, Sophia Zoungas, John Chalmers

**Affiliations:** 1 Department of Vascular Medicine, University Medical Centre Utrecht, PO Box 85500, 3508 GA Utrecht, the Netherlands; 2 The George Institute for Global Health, University of Sydney, Sydney, NSW Australia; 3 The George Institute for Global Health, Nuffield Department of Population Health, University of Oxford, Oxford, UK; 4 Julius Centre for Health Sciences and Primary Care, University Medical Centre Utrecht, Utrecht, the Netherlands; 5 The Royal Melbourne Hospital, University of Melbourne, Melbourne, VIC Australia; 6 University of Sheffield and Sheffield Teaching Hospitals NHS Foundation Trust, Sheffield, UK; 7 Istituto Auxologico Italiano, University of Milano-Bicocca, Milan, Italy; 8 Hôpital Bichat-Claude Bernard and Université Paris 7, Paris, France; 9 International Centre for Circulatory Health, Imperial College, London, UK

**Keywords:** Glycaemic target, Hypoglycaemia, Net benefit, Personalised medicine, Type 2 diabetes, Vascular complications

## Abstract

**Aims/hypothesis:**

Intensive glucose control reduces the risk of vascular complications while increasing the risk of severe hypoglycaemia at a group level. We sought to estimate individual beneficial and adverse effects of intensive glucose control in patients with type 2 diabetes.

**Methods:**

We performed a post hoc analysis of the Action in Diabetes and Vascular Disease: Preterax and Diamicron MR Controlled Evaluation (ADVANCE) trial, a randomised controlled trial evaluating standard vs intensive glucose control (HbA_1c_ target ≤6.5% [48 mmol/mol]). In 11,140 participants, we estimated the individual 5 year absolute risk reduction (ARR) for the composite outcome of major micro- and macrovascular events and absolute risk increase (ARI) for severe hypoglycaemia for intensive vs standard glucose control. Predictions were based on competing risks models including clinical characteristics and randomised treatment.

**Results:**

Based on these models, 76% of patients had a substantial estimated 5 year ARR for major vascular events (>1%, 5 year number-needed-to-benefit [NNTB_5_] <100) and 1% had a small ARR (<0.5%, NNTB_5_ >200). Similarly, 36% of patients had a substantial estimated ARI for severe hypoglycaemia (5 year number-needed-to-harm [NNTH_5_] <100) and 29% had a small ARI (NNTH_5_ >200). When assigning similar or half the weight to severe hypoglycaemia compared with a major vascular event, net benefit was positive in 85% or 99% of patients, respectively. Limiting intensive treatment to the 85% patient subgroup had no significant effect on the overall incidence of major vascular events and severe hypoglycaemia compared with treating all patients.

**Conclusions/interpretation:**

Taking account of the effects of intensive glucose control on major micro- and macrovascular events and severe hypoglycaemia for individual patients, the estimated net benefit was positive in the majority of the participants in the ADVANCE trial. The estimated individual effects can inform treatment decisions once individual weights assigned to positive and adverse effects have been specified.

***Trial registration*:**

ClinicalTrials.gov NCT00145925

**Electronic supplementary material:**

The online version of this article (doi:10.1007/s00125-016-4082-5) contains peer-reviewed but unedited supplementary material, which is available to authorised users.

## Introduction

Type 2 diabetes mellitus is a growing worldwide health problem, with 592 million people predicted to be living with diabetes by 2035 [1]. Observational studies have shown a close relationship between hyperglycaemia and the risk of vascular complications [2–4]. Subsequently, randomised trials have demonstrated beneficial effects of intensive glucose control on the incidence of microvascular diseases, such as retinopathy and nephropathy [5, 6]. The reduction in risk of macrovascular events has been modest in the short and medium term and may take more time to accrue [7–9]. However, intensive glucose control is also associated with disadvantages, such as an approximately doubled risk of severe hypoglycaemia depending on the glucose-lowering treatment being received [6, 10]. Severe hypoglycaemia is, in turn, associated with a nearly threefold increased risk of premature death, although this association may not be causal [11].

The increased risk of death with intensive glucose control observed in the Action to Control Cardiovascular Risk in Diabetes (ACCORD) trial has fuelled debate about whom to treat and what glycaemic target to use [12]. Current guidelines recommend a patient-centred approach, with consideration of the patient’s risk of hypoglycaemia, but offer few tools to identify patients for whom a stricter glycaemic target is likely to be worthwhile [13, 14]. Indeed, individual patients will have different chances to benefit from treatment and, similarly, a varied risk of experiencing the negative effects of treatment. For example, the anticipated risk reduction of vascular complications by intensive glucose control for individual patients depends on the estimated risk of vascular events [15]. Similarly, the susceptibility to severe hypoglycaemia is variable and patients will be affected by treatment differently [11]. Further, the appraisal of the potential short-term negative effects and long-term beneficial vascular effects of treatment will vary between patients. Thus, for some individuals the disadvantages of targeting near normal glucose levels might offset treatment benefits [16, 17].

In the present study, we aimed to estimate the beneficial and adverse effects of intensive glucose management, in terms of risk reduction for major vascular events and risk increase for severe hypoglycaemia, for individual patients with type 2 diabetes from the ADVANCE trial.

## Methods

The design, rationale and outcomes of the ADVANCE trial have been described elsewhere [18, 19]. Briefly, the trial was a factorial randomised controlled trial that evaluated the effect of intensive glucose control and BP lowering in individuals diagnosed with type 2 diabetes, aged ≥55 years, from 215 collaborating centres in 20 countries in Asia, Australasia, Europe and North America. Eligible individuals had a history of micro- or macrovascular disease, or at least one risk factor for vascular disease. There were no HbA_1c_ or BP criteria for inclusion. Intensive glucose control was defined as the use of gliclazide (modified release) plus other drugs, as required, to achieve an HbA_1c_ value of ≤6.5% (48 mmol/mol). The target HbA_1c_ value for standard glucose control was defined by local guidelines.

The endpoints considered in the current study were major vascular events (the original primary outcome comprising major micro- and macrovascular events) and severe hypoglycaemia. Macrovascular events included death from cardiovascular causes, myocardial infarction or stroke. Microvascular events included new or worsening nephropathy (i.e. development of macroalbuminuria [defined as urinary albumin/creatinine ratio (UACR) >300 μg/mg or doubling of serum creatinine to ≥200 mmol/l], the need for renal replacement therapy or death due to renal disease) or retinopathy (i.e. development of proliferative retinopathy, macular oedema or diabetes-related blindness, or the use of retinal photocoagulation therapy).

Hypoglycaemia was defined as a plasma glucose level of <2.8 mmol/l or the presence of typical symptoms and signs of hypoglycaemia without another apparent cause. Patients with transient dysfunction of the central nervous system who required help from another person were considered to have severe hypoglycaemia. An independent endpoint adjudication committee, unaware of the group assignments, reviewed source documentation for all suspected primary endpoints and deaths.

Data were missing in 4.5% of participants for UACR and in <1% for all other variables. Missing data were imputed by single imputation methods using predictive mean matching [20]. Approval for the trial was obtained from the institutional ethics committee of each centre and all participants provided written informed consent.

### Model derivation

We developed two Fine and Gray competing risks models for the prediction of major vascular events and severe hypoglycaemia based on the same set of demographic and clinical characteristics together with treatment status (standard vs intensive treatment) [21]. Death was considered as a competing event. The pre-specified predictors at baseline were: sex, age, diabetes duration, untreated and treated systolic BP, randomised BP-lowering treatment allocation, current smoking, HbA_1c_, non-HDL-cholesterol, waist circumference, UACR, estimated GFR (eGFR), history of macrovascular disease, history of microvascular disease, geographical region, educational attainment and treatment status. eGFR was calculated by the Chronic Kidney Disease Epidemiology Collaboration (CKD-EPI) equation [22]. Participants were classified into three regions of origin: established market economies (EME; reference category), Asia and Eastern Europe [23]. Educational attainment was defined according to age at completion of the highest level of formal education and categorised as less (age ≤15 years, approximately corresponding to age of completion of junior high school education in most regions) or more (age ≥16 years). Restricted cubic splines were used to assess the linearity assumption for continuous predictors [24, 25]. As a result, eGFR was included both as a linear and squared term and UACR was natural log transformed [26]. Potential interactions between treatment and estimated risk, and between treatment and baseline HbA_1c_ levels were considered for each outcome and included if the conditional likelihood ratio *p* value was <0.05.

The final models were used to calculate the risk of major vascular events and severe hypoglycaemia, with and without treatment, for every participant by fixing the treatment variable to standard and intensive treatment, respectively. The difference was the individual patient’s 5 year absolute risk reduction or increase (ARR or ARI), also expressed as 5 year number-needed-to-treat for one additional patient to benefit (NNTB_5_) or be harmed (NNTH_5_) [27]. A 5 year risk difference of >1% was considered substantial and a risk difference of <0.5% was considered small in this study. The model was fitted for the prediction of 5 year (median follow-up) risk.

Given the potential for an excess risk of death within 5 years with intensive glucose control [12], we repeated our analyses for an alternative outcome: the risk of death from any cause. Treatment interactions with HbA_1c_ and estimated mortality risk, as a proxy for patient frailty, were also considered.

### Assessment of model performance and internal validation

Calibration was assessed by plotting the observed 5 year cumulative incidence against the average predicted 5 year event risk within groups defined by the deciles of predicted risk. Discrimination was assessed at 5 years by the c-statistic accounting for right censoring [28]. Further, we assessed the amount of over-optimism by 100-fold bootstrap resampling to derive a shrinkage factor to uniformly shrink the model coefficients and to obtain an optimism-corrected c-statistic [24, 25].

### Distribution of individual treatment effect and net benefit

The distributions of estimated individual patient 5 year ARR of major vascular events and 5 year ARI of severe hypoglycaemia were displayed in histograms. Net benefit was calculated as the difference between ARR and ARI for individual patients when assuming either half or similar weight for severe hypoglycaemia. Subsequently, we ranked patients based on their predicted net benefit from largest to smallest and showed the overall effect of treating a gradually increasing proportion of patients on the incidence of major vascular events and severe hypoglycaemia.

Further, we evaluated whether selective treatment strategies based on predicted treatment effects could result in a more favourable trade-off between the reduction of major vascular events, increase in severe hypoglycaemia and number of patients treated, compared with treating everyone (treating none was not considered to be clinically acceptable). Therefore, based on the prediction algorithms, we selectively collected patients with an estimated positive net effect from the intervention group and patients with a null or negative net benefit from the control group. Incidence rates in these newly assembled artificial populations were compared with incidence rates in the intensive glucose control arm (the reference population). Statistical analyses were conducted in R, version 3.1.1 (R Development Core Team, Vienna, Austria).

## Results

### Model derivation and performance

**Fig. 1:**
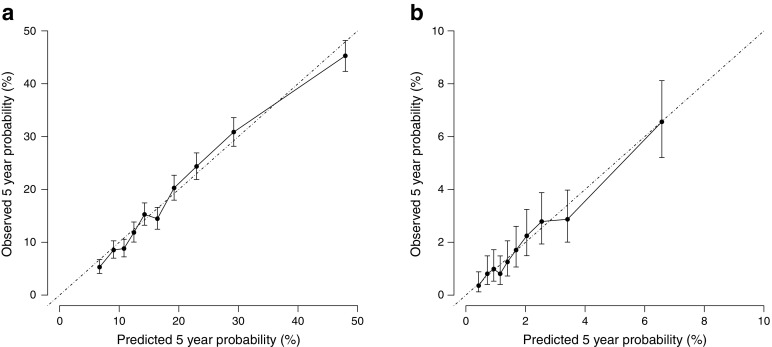
Calibration plots. Predicted vs observed 5 year risk of (**a**) major vascular events and (**b**) severe hypoglycaemia in ADVANCE participants (*N* = 11,140). Data is presented as mean ± 95% CI

The baseline characteristics of the ADVANCE participants are shown in Table 1 and the use of glucose-lowering drugs in the electronic supplementary material (ESM) Table 1. During a median follow-up of 5.0 years, 2125 major micro- and macrovascular events and 399 non-cardiovascular deaths occurred. From these data a risk prediction algorithm for adverse events was produced (Table 2). Interactions between intensive treatment and the estimated risk of major vascular events and HbA_1c_ level were not significant (*p* > 0.6). The risk score showed good calibration (Fig. 1a) and moderate discrimination (optimism-adjusted c-statistic = 0.68 [95% CI 0.67, 0.70]).

**Table 1:** Baseline characteristics of the ADVANCE trial population

Characteristic	Total population (*N* = 11,140)
Female, *n* (%)	4733 (42)
Age (years)^a^	66 ± 6
Duration of diabetes (years)^a^	8 ± 6
History of microvascular disease, *n* (%)	1155 (10)
History of macrovascular disease, *n* (%)	3590 (32)
Region	
EME, *n* (%)	4862 (44)
Eastern Europe, *n* (%)	2142 (19)
Asia, *n* (%)	4136 (37)
Blood glucose control	
Fasting blood glucose (mmol/l)^a^	8.5 ± 2.8
Serum HbA_1c_ concentration (%)^a^	7.5 ± 1.6
Serum HbA_1c_ concentration (mmol/mol)^a^	58 ± 18
Other risk factors	
Systolic BP (mmHg)^a^	145 ± 22
Diastolic BP (mmHg)^a^	81 ± 11
History of treated hypertension, *n* (%)	7655 (69)
Non-HDL-cholesterol (mmol/l)^a^	3.9 ± 1.1
Triacylglycerol (mmol/l)^b^	1.6 (1.2–2.3)
UACR (mg/mmol)^b^	1.7 (0.8–4.5)
eGFR (ml/min/1.73 m^2^)^b^	75 (62–89)
Current smoking, *n* (%)	1682 (15)
Waist circumference (cm)^a^	99 ± 13
Educational attainment^c^, *n* (%)	7121 (64)

Data is presented as %, ^a^mean ± SD or ^b^median with interquartile range

^c^Age ≥16 years at completion

**Table 2:** Details of the Fine and Gray competing risks proportional hazards model for the estimation of major vascular events

Variable	Coefficient^a^	sHR	95% CI	*p* value
Glucose treatment allocation (intensive vs standard)	−0.0992	0.90	0.83,0.98	0.021
Sex (women vs men)	−0.3548	0.70	0.63, 0.77	<0.001
Age (per 1 year)	0.0099	1.01	1.00, 1.02	0.009
Duration of diabetes (per 1 year)	0.0279	1.03	1.02, 1.04	<0.001
Systolic BP if untreated (per 1 mmHg)	0.0039	1.00	1.00, 1.01	0.000
Systolic BP if treated (per 1 mmHg)	0.0050	1.01	1.00, 1.01	<0.001
Non-HDL-cholesterol (per 1 mmol/l)	0.0429	1.04	1.01, 1.08	0.022
HbA_1c_ (per 1%)	0.1189	1.13	1.10, 1.16	<0.001
UACR (per 1 mg/mmol log_e_)	0.1625	1.18	1.14, 1.22	<0.001
eGFR (per 1 ml/min increase)	−0.0255	0.97	0.96, 0.99	<0.001
eGFR squared (per 1 ml/min^2^)	0.0001	1.00	1.00, 1.00	0.010
Waist circumference (per 1 cm)	−0.0003	1.00	1.00, 1.00	0.880
Smoking (current vs never or former)	0.0032	1.00	0.88, 1.14	0.960
History of microvascular disease (yes vs no)	0.4738	1.62	1.42, 1.86	<0.001
History of macrovascular disease (yes vs no)	0.3180	1.38	1.26, 1.51	<0.001
Educational attainment (≥16 years at completion of education)	−0.2309	0.79	0.72, 0.87	<0.001
Region				
Eastern Europe	0.0344	1.04	0.90, 1.19	0.610
Asia	0.2879	1.34	1.19, 1.51	<0.001
BP treatment allocation (perindopril/indapamide vs placebo)	−0.0822	0.92	0.84, 1.00	0.056

5 year major vascular event risk (%) = (1 − S_0_(5)^exp(A-1.6641)^) × 100%. Where S_0_(5) = 0.8363 (the 5 year baseline survival) and A is the sum, over all variables in the model, of the patient’s specific value × the corresponding coefficient

^a^Coefficients were penalised by a shrinkage factor of 0.979 to increase external validity, whereas unbiased HRs and statistics were derived from an unpenalised Fine and Gray model

To convert values for HbA_1c_ in % to mmol/mol, subtract 2.15 and multiply by 10.929

sHR, subdistribution hazard ratio

**Fig. 2:**
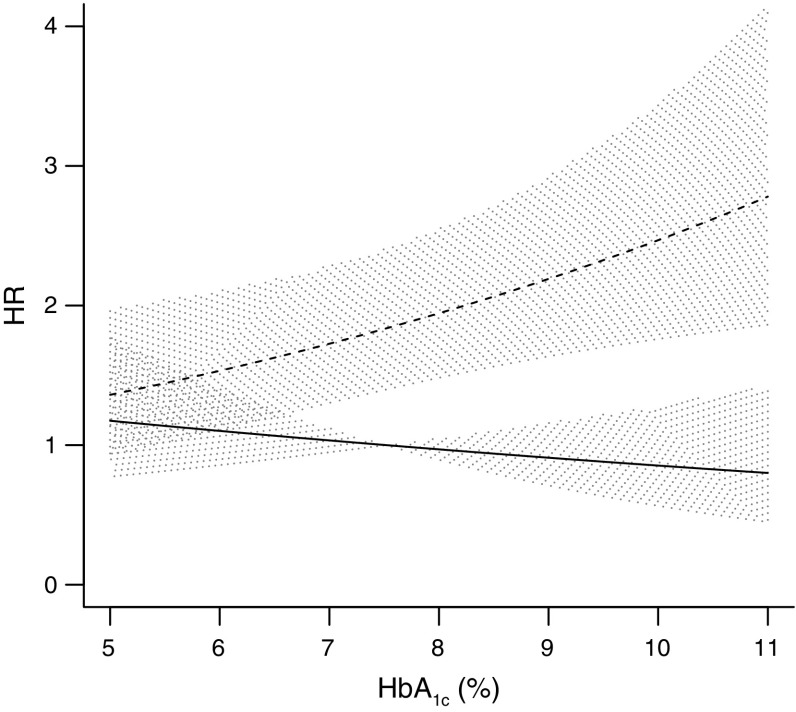
Graphical representation of the interaction between glucose-lowering treatment and baseline HbA_1c_ on the risk of severe hypoglycaemia. HbA_1c_ is expressed continuously on the *x*-axis. The *y*-axis shows the risk of severe hypoglycaemia under standard and intensive glucose control relative to a person with an HbA_1c_ of 7.5% (the mean) on standard glucose control. To convert values for HbA_1c_ in % to mmol/mol, subtract 2.15 and multiply by 10.929. Solid line, standard glucose control with 95% CI; dashed line, intensive glucose control with 95% CI

During follow-up, 231 first severe hypoglycaemia episodes occurred. The risk prediction algorithm for severe hypoglycaemia is shown in Table 3. The interaction between treatment and the estimated risk of hypoglycaemia was not significant (*p* = 0.8). By contrast, the interaction with baseline HbA_1c_ indicated significantly higher risks of hypoglycaemia across increasing levels of HbA_1c_ only in those assigned to intensive glucose control (*p* = 0.048) (Fig. 2). Model calibration (Fig. 1b) was good and discrimination was moderate (optimism-adjusted c-statistic 0.68 [95% CI 0.62, 0.74]).

**Table 3:** Details of the Fine and Gray competing risks proportional hazards model for the estimation of severe hypoglycaemia

Variable	Coefficient^a^	sHR	95% CI	*p* value
Glucose treatment allocation (intensive vs standard)^b^	−0.6808	0.46	0.12, 1.87	0.280
Sex (women vs men)	0.0661	1.08	0.80, 1.45	0.630
Age (per 1 year)	0.0242	1.03	1.01, 1.05	0.016
Duration of diabetes (per 1 year)	0.0278	1.03	1.01, 1.05	0.002
Systolic BP if untreated (per 1 mmHg)	0.0021	1.00	1.00, 1.01	0.520
Systolic BP if treated (per 1 mmHg)	0.0015	1.00	0.99, 1.01	0.630
Non-HDL-cholesterol (per 1 mmol/l)	−0.0620	0.93	0.82, 1.06	0.290
HbA_1c_ (per 1%)	−0.0566	0.94	0.79, 1.11	0.450
HbA_1c_ by treatment (add if on intensive treatment, per 1%)^b^	0.1622	1.20	1.00, 1.44	0.048
UACR (per 1 mg/mmol log_e_)	−0.0089	0.99	0.90, 1.09	0.830
eGFR (per 1 ml/min increase)	−0.0247	0.97	0.94, 1.01	0.097
eGFR squared (per 1 ml/min^2^)	0.0001	1.00	1.00, 1.00	0.520
Waist circumference (per 1 cm)	−0.0130	0.99	0.97, 1.00	0.031
Smoking (current vs never or former)	0.1764	1.22	0.83, 1.80	0.310
History of microvascular disease (yes vs no)	0.6043	1.98	1.38, 2.84	<0.001
History of macrovascular disease (yes vs no)	0.1775	1.22	0.92, 1.61	0.160
Educational attainment (≥16 years at completion of education)	−0.3064	0.71	0.54, 0.93	0.014
Region				
Eastern Europe	−0.6643	0.47	0.29, 0.77	0.003
Asia	−0.0592	0.94	0.66, 1.33	0.710
BP treatment allocation (perindopril/indapamide vs placebo)	0.1955	1.25	0.96, 1.62	0.098

5 year severe hypoglycaemia risk (%) = (1 − S_0_(5)^exp(A+1.1537)^) × 100%. Where S_0_(5) = 0.9845 (the 5 year baseline survival) and A is the sum, over all variables in the model, of the patient’s specific value × the corresponding coefficient

^a^Coefficients were penalised by a shrinkage factor of 0.886 to increase external validity, whereas unbiased HRs and statistics were derived from an unpenalised Fine and Gray model

^b^Needs to be combined with the interaction term to estimate effect of intensive glucose control at specific HbA_1c_ value (e.g. coefficient = −0.6808 + HbA_1c_ (%) × 0.1622)

To convert values for HbA_1c_ in % to mmol/mol, subtract 2.15 and multiply by 10.929

sHR, subdistribution hazard ratio

A complementary model for risk of death was developed based on 1031 deaths from any cause that occurred during follow-up (ESM Table 2). Treatment interactions with estimated risk and HbA_1c_ were not significant (*p* > 0.3). The risk score showed good calibration (ESM Fig. 1) and moderate discrimination (optimism-adjusted c-statistic 0.72 [95% CI 0.71, 0.75]).

### Distribution of treatment effects

**Fig. 3:**
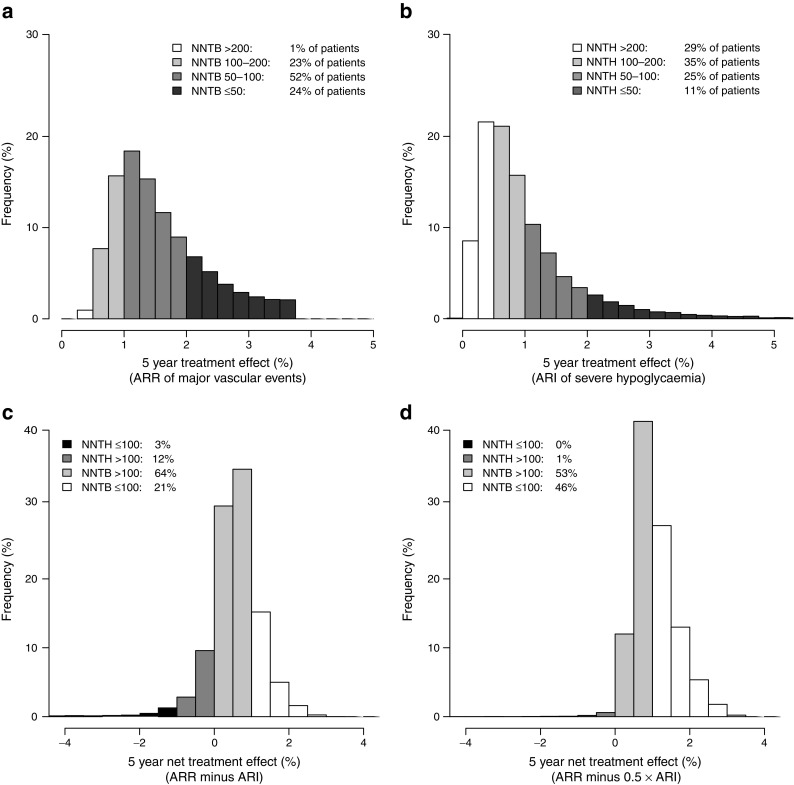
Distribution of individual patient treatment effects of intensive glucose control for (**a**) 5 year ARR for major vascular events and (**b**) ARI for severe hypoglycaemia, and net treatment effect assigning (**c**) half the weight or (**d**) similar weight to severe hypoglycaemia

The overall 5 year ARR for major vascular events was 1.3% (NNTB_5_=74) and the overall ARI for severe hypoglycaemia was 1.1% (NNTH_5_=87). Using the individual prediction algorithms, the estimated treatment effect on major vascular events and severe hypoglycaemia was calculated for each participant (see Text box for an example). In ADVANCE, 76% of participants had a substantial estimated 5 year ARR for major vascular events due to treatment (NNTB_5_ <100) and 1% of participants had a small ARR (NNTB_5_ >200). The proportion of patients with an intermediate ARR (NNTB_5_ 100–200) was 23% (Fig. 3a). With respect to the effect of intensive glucose control on severe hypoglycaemia, 36% of participants had a substantial predicted ARI (NNTH_5_ <100), whereas 29% had a small predicted ARI (NNTH_5_ >200). The proportion of patients with an intermediate ARI for severe hypoglycaemia (NNTH_5_ 100–200) was 35% (Fig. 3b).

**Table:** 

Estimated 5 year ARR for major vascular events and ARI for severe hypoglycaemia with intensive glucose control for a specific ADVANCE participant
A 62-year-old male patient from an EME country with a 7 year history of diabetes who has macrovascular disease, an HbA_1c_ of 6.7% (50 mmol/mol), a treated BP of 158/93 mmHg, a non-HDL-cholesterol level of 3.8 mmol/l, an eGFR of 79 ml/min and a UACR of 4.8 mg/mmol: • 5 year ARR for major vascular events = 2.0% (individual NNTB_5_ = 49) • 5 year ARI for severe hypoglycaemia = 0.5% (individual NNTH_5_ = 196)

Altogether, 99% (Fig. 3c) or 85% (Fig. 3d) of patients had a positive net benefit of treatment when assigning half or similar weight to severe hypoglycaemia compared with major vascular events, respectively. Assigning less than half the weight to severe hypoglycaemia resulted in a net positive effect for all participants. The characteristics of patients with net positive and negative treatment effects are summarised in ESM Table 3. In addition, the predicted absolute risk of death was lower with intensive treatment in all participants, irrespective of their estimated mortality risk or HbA_1c_ level.

### Group-level effects and clinical implications

**Fig. 4:**
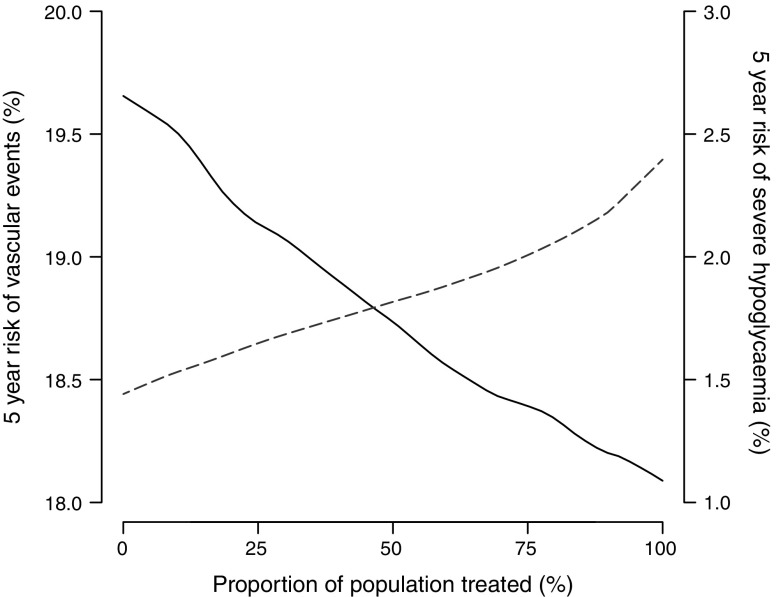
Overall effects of selective intensive glucose control treatment on the 5 year incidence of major vascular events and severe hypoglycaemia. The *x*-axis shows the proportion of patients treated. Patients were ranked according to their estimated net benefit (largest to smallest), e.g. the first 85% of patients had an estimated positive individual net effect. The left *y*-axis shows the 5 year risk of major vascular events (solid line) and the right *y*-axis shows the 5 year risk of severe hypoglycaemia (dashed line)

**Fig. 5:**
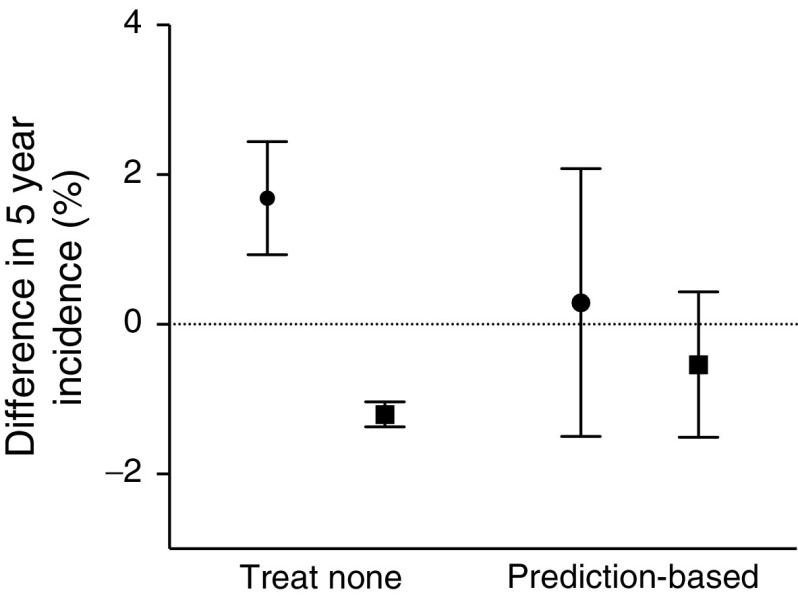
Overall effect of selective prediction-based treatment. 5 year effect on the incidence of major vascular events (ARR) and 5 year effect on the incidence of severe hypoglycaemia (ARI) compared with treating everyone (dotted line). The ‘prediction-based’ sample comprises patients with a positive estimated net benefit from the intervention group and patients with a null or negative estimated net benefit from the control group. Circle, major vascular events; square, severe hypoglycaemia. Error bars represent 95% CI

The overall effect of treating a proportion of patients ranked according to net benefit is shown in Fig. 4. Increasing the proportion of patients treated resulted in a lower incidence of major vascular events and higher incidence of severe hypoglycaemia. The observed overall effects of selective prediction-based treatment strategies are shown in Fig. 5. The selective strategy encompassed intensive treatment of 85% of patients with a predicted positive net benefit and standard glucose control for the 15% of patients with a predicted negative net benefit. This strategy was associated with a small non-significant increase of 0.3% (95% CI −3.7%, 4.3%) in the incidence of major vascular events compared with treating everyone. The incidence of severe hypoglycaemia simultaneously decreased non-significantly by 0.5% (95% CI −1.4%, 2.4%).

## Discussion

The present analysis using the ADVANCE trial demonstrates that patient-specific characteristics at baseline can be used to quantify the anticipated individual effects of intensive vs standard glucose control in terms of risk reduction for major vascular events and risk increase for severe hypoglycaemia. The estimated net benefit was positive in the majority of patients, depending upon the individual weight assigned to the beneficial and adverse effects of treatment.

The ADVANCE trial was designed to evaluate the efficacy of gliclazide-based intensive glucose control in a broad sample of patients with type 2 diabetes. The strategy of intensive treatment yielded an overall 10% relative risk reduction of the primary composite outcome of major micro- and macrovascular events. The effect was largely driven by a reduction in renal events by 21%, whereas the reduction in major cardiovascular events was only 6% [29]. However, meta-analyses substantiate the claim that intensive glucose control provides modest but significant protection from macrovascular disease [7, 8]. At the same time, intensive glucose control in ADVANCE was associated with a more than doubled risk of severe hypoglycaemia, although the average incidence was lower compared with similar trials [6]. A recent Cochrane review summarised absolute effects of intensive treatment and showed that the average NNTB_5_ ranged from 32 to 142 for microalbuminuria and from 117 to 150 for myocardial infarction. In addition, the average NNTH_5_ ranged from 15 to 52 for intensive glucose control [6]. However, these estimates of beneficial and harmful effects are the averages for entire study populations and are not informative of the treatment effect for individual patients [15]. Therefore, the latest joint statement from the ADA/EASD stressed the importance of a patient-centred approach including the consideration of diabetes duration, history of macrovascular disease and the individual’s hypoglycaemia risk [30]. The present study aimed to provide further details on the potential benefits and harms of treatment intensification for individual patients.

We show that prediction models based on multiple characteristics can quantify the estimated beneficial effects on major vascular events and adverse effects on severe hypoglycaemia of a gliclazide-based regime of intensive glucose control in individual patients. At a group level, treating a larger proportion of patients decreased the incidence of major vascular events and increased the incidence of severe hypoglycaemia. However, this is not informative of the risk to benefit ratio of intensive glucose control at a patient-specific level. Despite shared risk factors for both outcomes, they did vary in strength and produced variations in net estimated treatment effect for individual patients. Further, we observed a different relationship between baseline HbA_1c_ level and risk of severe hypoglycaemia in patients assigned to standard treatment compared with intensive treatment. In patients receiving intensive glucose control, the risk of severe hypoglycaemia increased with higher levels of HbA_1c_, whereas risk slightly decreased in patients receiving standard therapy. A higher risk of hypoglycaemia in patients with poorer glycaemic control was also demonstrated in the ACCORD trial [31]. In ADVANCE, the increased risk was confined to the intensive treatment arm, probably representing the use of more complex glucose-lowering strategies, including more frequent use of insulin, in an attempt to achieve the lower glycaemic target in this group.

Due to the combined and multiplicative effect of multiple risk factors, no single characteristic could be used to identify patients with a positive effect. Therefore, we developed a risk score incorporating multiple patient-specific variables to tailor the assessment of the risks and benefits of treatment for the individual patient. For some patients, the benefits of intensive glucose control will largely offset treatment disadvantages (see Text box). On the other hand, for patients with a smaller or absent net benefit of intensive treatment a less stringent treatment target might be considered. Further, individual treatment decisions are critically dependent on the relative appraisal of beneficial and adverse treatment effects. The present analysis indicated that the majority (85%) of patients in ADVANCE derived a net benefit from intensive glucose control when positive and negative effects were assumed to be equally important. However, the comparison of the immediate risk of severe hypoglycaemia in terms of clinical importance and relevance with, for example, the long-term prevention of end stage renal disease is difficult for both patients and clinicians. Some patients are likely to attach greater weight to avoiding severe hypoglycaemia in relation to, for example, their occupation or the presence of hypoglycaemia unawareness. At a group level, a selective treatment strategy aimed at treating the 85% patient subgroup with an estimated net benefit had no effect on the overall incidence of major vascular events or severe hypoglycaemia. Thus, selective treatment according to conflicting risks may benefit the individual patient while achieving similar population level effects compared with treating everyone.

Importantly, we showed that intensive glucose control was not associated with the risk of death from any cause. There was a similar, albeit non-significant, relative risk reduction of death with intensive treatment across different levels of estimated mortality risk as evaluated by the continuous interaction terms between treatment and risk [32, 33]. The interaction of intensive treatment with HbA_1c_ level on risk of death, as reported in a post hoc analysis of the ACCORD trial, was not significant in this study [34]. Hence, the present analyses did not find any evidence of an excess risk of death with intensive glucose control, not even for the potentially most vulnerable patients who are at highest risk of death.

Some limitations need to be considered with respect to this study; first, follow-up data under randomised therapies were only available for a median of 5.0 years, while treatment is often lifelong. In addition, treatment targets for participants were fixed at study enrolment whereas in clinical practice treatment goals are often revised at repeated outpatient visits. Nevertheless, in ADVANCE the intensive treatment target was gradually reached over a period of 3 years. Hence, this paper provides estimates of the individual benefits and harms of adhering to a specific treatment target in the longer term. Second, the present comparison used a composite outcome of major beneficial effects, comprising individual components with a potentially different clinical impact. However, clinical usefulness and interpretation will benefit from such a summary outcome of positive vascular effects. For example, the development of macroalbuminuria might go unnoticed to a patient but does confer a three to fivefold increased risk of (cardiovascular) mortality [35]. Similarly, we focused on the main adverse effect of treatment; effects on weight change were not incorporated, yet earlier concerns about weight gain were largely refuted for the gliclazide-based regimen used in ADVANCE [36]. Also, mild hypoglycaemia (which can, for example, result in avoiding exercise and increased worry about hypoglycaemia that affects daily life) was not incorporated into this study since it is not related to the risk of vascular events or death [11, 37, 38]. Third, the analyses and estimates related to this study apply to those patients who were eligible for inclusion and were treated with a gliclazide-based regimen. At the end of follow-up the most frequently prescribed drugs were gliclazide, metformin and insulin. Although the use of sulfonylureas is decreasing, they are firmly embedded in the current guidelines [30, 39]. Further, novel therapies such as incretin-based drugs and sodium-glucose cotransporter-2 inhibitors may demonstrate different safety profiles and result in different risk to benefit ratios. Fourth, in the absence of available external risk algorithms for the outcomes of interest, we derived new prediction models that are likely to perform optimistically in the sample from which they were derived [24]. Therefore, we evaluated the amount of over-optimism and provided adjusted effect estimates and performance measures. Finally, individual estimates are accompanied by larger uncertainty margins. However, once the causal effects of treatment have been established at a group level, the point estimate is the most likely approximation of the true effect for the individual patient and is most useful to inform medical decisions.

In conclusion, the individual effects of intensive glucose control in terms of reducing the risk of major vascular events and increasing the risk of severe hypoglycaemia can be quantified using a multivariable risk algorithm. The estimated net benefit was positive in the majority of patients in the ADVANCE trial, with the percentage benefiting depending upon the individual weight assigned to the beneficial and adverse effects of treatment.

## Electronic supplementary material

Below is the link to the electronic supplementary material.

ESM (PDF 144 kb)

## Abbreviations

ADVANCE: Action in Diabetes and Vascular Disease: Preterax and Diamicron MR Controlled Evaluation
ACCORD: Action to Control Cardiovascular Risk in Diabetes
ARI: Absolute risk increase
ARR: Absolute risk reduction
CKD-EPI: Chronic Kidney Disease Epidemiology Collaboration
eGFR: Estimated GFR
EME: Established market economies
NNTB_5_: 5 year number-needed-to-benefit
NNTH_5_: 5 year number-needed-to-harm
UACR: Urinary albumin/creatinine ratio
