# Supplementary material for: Estimation of individual beneficial and adverse effects of intensive glucose control for patients with type 2 diabetes
Source: Diabetologia. 2016 Sep 1;59(12):2603–12. doi: 10.1007/s00125-016-4082-5 (PMC6518074; doi:10.1007/s00125-016-4082-5)

Electronic Supplementary Material

Estimation of individual beneficial and adverse effects of intensive glucose control for patients with type 2 diabetes, Van der Leeuw et al.

Table 1. The use of glucose-lowering drugs at baseline and end of follow-up.

Glucose-lowering drug	Baseline		End of follow-up	
	**Intensive control** (n=5571)	**Standard control** (n=5569)	**Intensive control** (n=4828)	**Standard control** (n=4741)
Gliclazide (modified-release), %*	7.6	8	90.5	1.6
Other sufonylurea, %	64.2	63.1	1.9	57.1
Metformin, %	61	60.2	73.8	67
Thiazolidinedione, %	3.6	3.7	16.8	10.9
Acarbose, %	9.2	8	19.1	12.6
Glinide, %	1.8	1.5	1.2	2.8
Any oral hypoglycaemic drug, %	91.3	90.6	93.7	84.4
Insulin, %	1.5	1.4	40.5	24.1
None, %	8.7	9.4	1.5	6.4

*The use of gliclazide (modified release) at baseline in the intensive-control group is reported for the first (registration) visit; at the randomization visit, 99% of patients in this group were given the drug

Table 2. Details of the final Cox proportional hazards model for the estimation of death from any cause.

	S_0 (5) = 0.9294			
**Variable**	**Coefficient**¶	**sHR**	**95% CI**	***p*-value**
Glucose treatment allocation (intensive vs. standard)	-0.0531	0.95	0.84 - 1.07	0.383
Sex (women vs. men)	-0.5710	0.56	0.48 - 0.64	<0.001
Age (per 1 year)	0.0736	1.08	1.07 - 1.09	<0.001
Duration of diabetes (per 1 year)	0.0201	1.02	1.01 - 1.03	<0.001
Systolic blood pressure if untreated (per 1 mmHg)	0.0010	1.00	1.00 - 1.00	0.518
Systolic blood pressure if treated (per 1 mmHg)	0.0017	1.00	1.00 - 1.00	0.245
Non HDL cholesterol (per 1 mmol/l)	0.0641	1.07	1.01 - 1.13	0.020
HbA_{1c} (per 1%)	0.0880	1.09	1.05 - 1.14	<0.001
UACR (per 1 mg/mmol $\log_e$)	0.1369	1.15	1.10 - 1.20	<0.001
eGFR (per 1 ml/min increase)	-0.0316	0.97	0.95 - 0.99	<0.001
eGFR squared (per 1 ml/min squared)	0.0002	1.00	1.00 - 1.00	0.017
Waist circumference (per 1 cm)	0.0039	1.00	1.00 - 1.01	0.152
Smoking (current vs. never or former)	0.3051	1.37	1.15 - 1.63	<0.001
History of microvascular disease (yes vs. no)	0.3453	1.42	1.20 - 1.70	<0.001
History of macrovascular disease (yes vs. no)	0.3711	1.46	1.29 1.66	<0.001
Educational attainment (>15y at completion of education)	-0.3389	0.71	0.62 - 0.81	<0.001
Region				
Eastern Europa	0.4225	1.54	1.30 1.83	<0.001
Asia	0.1116	1.12	0.95 1.33	0.188
BP treatment allocation (perindopril/indapamide vs. placebo)	-0.1042	0.90	0.80 - 1.02	0.087

$S_0(5)$= 5-year baseline survival, sHR: subdistribution hazard ratio, HDL: high density lipoprotein, UACR: urinary albumin/creatinin ratio, eGFR: estimated glomerular filtration rate (CKD-EPI equation). ¶ Coefficients were penalized by a shrinkage factor of 0.975 to increase external validity, whereas unbiased HRs and statistics were derived from an unpenalised Fine and Gray model.
5-year death risk (%) = $(1 - S_0(5)^{\exp(A-5.255)})$ x 100%. A is the sum, over all variables in the model, of the patient's specific value times the corresponding coefficient.

Table 3. Baseline characteristics of the ADVANCE population after stratification to net positive and negative treatment effect.

**Characteristic**	**Net positive effect*** (n=9,488)	**Net negative effect*** (n=1,652)
Female, n(%)	3526 (38)	1107 (67)
Age (years)[a]	65 (6)	68 (6)
Duration of diabetes (years)[a]	8 (6)	10 (7)
History of microvascular disease, n(%)	854 (9)	301 (18)
History of macrovascular disease, n(%)	3179 (34)	411 (25)
Region		
Established market economies, n(%)	3853 (41)	1003 (61)
Eastern Europe, n(%)	2094 (22)	48 (3)
Asia, n(%)	3535 (37)	601 (36)
Blood glucose control		
Fasting blood glucose (mmol/l)[a]	8.2 (2.6)	9.9 (3.4)
Serum haemoglobulin A_{1c} concentration (%)[a]	7.3 (1.4)	8.8 (1.9)
Serum haemoglobulin A_{1c} concentration (mmol/mol)[a]	56 (15)	73 (21)
Other risk factors		
Systolic blood pressure (mmHg)[a]	146 (21)	141 (22)
Diastolic blood pressure (mmHg)[a]	81 (11)	77 (11)
History of treated hypertension, n(%)	6855 (72)	800 (48)
Non HDL-cholesterol (mmol/l)[a]	4.0 (1.2)	3.7 (1.0)
Triacylglycerol (mmol/l)[b]	1.6 (1.2 - 2.3)	1.6 (1.2 - 2.2)
Urinary albumin:creatinine ratio (mg/mmol)[b]	1.8 (0.9 - 4.7)	1.1 (0.1 - 2.7)
eGFR (mL/min/1.73m^2)[b]	76 (63 - 89)	67 (56 - 81)
Current smoking, n(%)	1416 (15)	266 (16)
Waist circumference (cm)[a]	100 (13)	92 (12)
Educational attainment (age >15 y at completion)[a]	6416 (68)	705 (43)

[a] mean ± standard deviation; [b] median with interquartile range; eGFR: estimated glomerular filtration rate by CKD-EPI formula; HDL: high density lipoprotein. *Net positive/negative treatment effect: ARR of major vascular events > or ≤ ARI of severe hypoglycaemia (assuming equal weights)

Figure 1. **Calibration plot**. Predicted versus observed 5-year risk of death from any cause in ADVANCE participants.

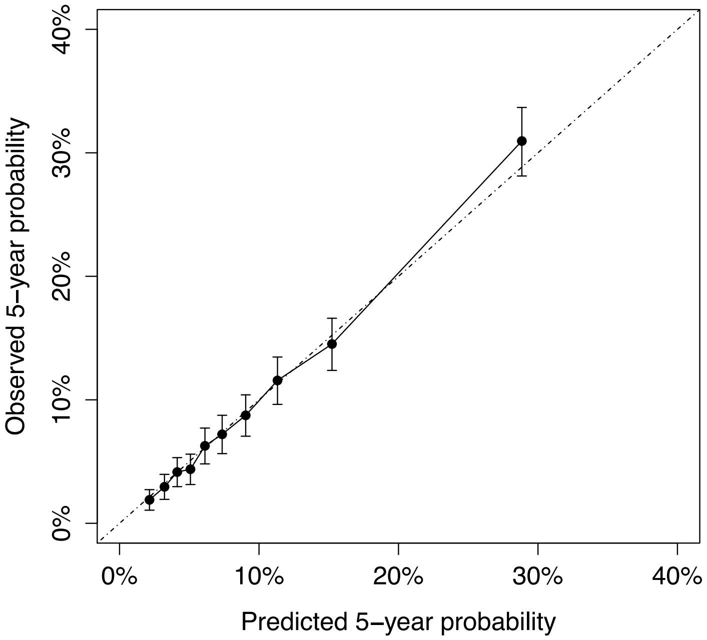

Supplement: Supplementary file 1 — (PDF 144 kb) [file 125_2016_4082_MOESM1_ESM.pdf]
